# Seroprevalence of Q fever among human and animal in Iran; A systematic review and meta-analysis

**DOI:** 10.1371/journal.pntd.0005521

**Published:** 2017-04-10

**Authors:** Ashraf Mohabbati Mobarez, Fahimeh Bagheri Amiri, Saber Esmaeili

**Affiliations:** 1 Department of Bacteriology, Faculty of Medical Sciences, Tarbiat Modares University, Tehran, Iran; 2 Department of Epidemiology, Faculty of Veterinary Medicine, University of Tehran, Tehran, Iran; 3 Urology and Nephrology Research Center, Shahid Beheshti University of Medical Sciences, Tehran, Iran; 4 National Reference Laboratory of Plague, Tularemia and Q Fever, Research Centre for Emerging and Reemerging Infectious Diseases, Pasteur Institute of Iran, Akanlu, Kabudar-Ahang, Hamadan, Iran; Texas A&M University College Station, UNITED STATES

## Abstract

**Background:**

Q fever is a main zoonotic disease around the world. The aim of this meta-analysis was to estimate the overall seroprevalence of *Coxiella burnetii* among human and animal population in Iran.

**Methods:**

Major national and international databases were searched from 2005 up to August 2016. We extracted the prevalence of Q fever antibodies (IgG) as the main primary outcome. We reported the prevalence of the seropositivity as point and 95% confidence intervals.

**Results:**

The overall seroprevalence of IgG phase I and II antibodies of Q fever in human was 19.80% (95% CI: 16.35–23.25%) and 32.86% (95% CI: 23.80–41.92%), respectively. The herd and individual prevalence of *C*. *burnetii* antibody in goat were 93.42% (95% CI: 80.23–100.00) and 31.97% (95% CI: 20.96–42.98%), respectively. The herd and individual prevalence of Q fever antibody in sheep's were 96.07% (95% CI: 89.11–100.00%) and 24.66% (95% CI: 19.81–29.51%), respectively. The herd and individual prevalence of *C*. *burnetii* antibody in cattle were 41.37% (95% CI: 17.88–64.86%) and 13.30% (95% CI: 2.98–23.62%), respectively. Individual seropositivity of Q fever in camel and dog were 28.26% (95% CI: 21.47–35.05) and 0.55% (0.03–2.68), respectively.

**Conclusion:**

Seroprevalence of Q fever among human and domestic animals is considerable. Preventative planning and control of *C*. *burnetii* infections in Iran is necessary. Active surveillance and further research studies are recommended, to more clearly define the epidemiology and importance of *C*. *burnetii* infections in animals and people in Iran.

## Author summary

Q fever is a zoonotic diseases caused by a bacterium so called *Coxiella burn*etii. Domestic ruminants (primarily cattle, sheep and goats) are the most important reservoir of *C*. *burnetii* in the nature. Q fever is mostly asymptomatic in livestock and animals. Clinical manifestations of Q fever in humans includes asymptomatic, acute, chronic to fatigue syndrome. Acute Q fever is defined as primary infection with *C*. *burnetii*, and <60% of infected patients may be asymptomatic. Acute Q fever can manifest as a flu-like and self-limited illness. Chronic Q fever is accompanied with endocarditis and vascular infection which is fatal if untreated. The results of this meta-analysis showed the prevalence of IgG phase I and II antibodies of *C*. *burnetii* among human in Iran were 19.80% and 32.86%, respectively. The prevalence of Q fever antibodies in cattle, goat and sheep were 13.30%, 31.97% and 24.66% in Iran, respectively. Seroprevalence of Q fever among human and domestic animals is considerable. Preventative planning and control of *C*. *burnetii* infections in Iran is necessary. Active surveillance and further research studies are recommended, to more clearly define the epidemiology and importance of *C*. *burnetii* infections in animals and people in Iran.

## Introduction

Q fever is a zoonosis caused by the intracellular, gram negative bacterium *Coxiella burnetii*. *C*. *burnetii* is an extremely infectious pathogen [1]. The extremely high infectivity, the ability to withstand harsh environmental conditions, and the potential to cause severe disease in man, has deemed this organism to be considered as a biological terrorist agent. It has been listed as a Category B biological warfare agent by the Centre’s of Disease Control and Prevention [2,3].

*C*. *burnetii* infects people and a wide range of wild and domesticated animals. Within the environment, *C*. *burnetii* survives in arthropod hosts, such as ticks. From these hosts it can spread, and it primarily spreads into ruminants. Domestic ruminants (primarily cattle, sheep and goats) are the most important reservoir of *C*. *burnetii* in the nature. Q fever is mostly asymptomatic in livestock and animals, except in some cases, where causes abortion, stillbirth, endometritis or infertility. Infected animals shed *C*. *burnetii* into the environment in milk, colostrum, urine, vaginal discharges and especially in birth products [4,5]. High numbers of organisms exist in the amniotic fluids and placenta during birthing (e.g., 10^9^ bacteria/g placenta) [6]. *C*. *burnetii* can survive for long periods in the environment, and it is common for aerosols from infected herds to be carried by the wind and cause infection in humans. Q fever outbreaks could be directly connected to the speed and frequency of the wind [7]. Inhalation of infectious aerosol or contaminated dusts containing air-borne bacterium the major route of acquiring the disease in humans, so that a single inhaled organism may produce clinical illness. Nevertheless, the other routes of transmission of this infection to human are consumption of contaminated milks and dairy products, skin or mucosal contact, tick bites, blood transfusion, sexual transmission and embryo transfer [4,5,8].

Clinical manifestations of Q fever in humans includes acute, chronic to fatigue syndrome. The main characteristic of Q fever is its clinical polymorphism. Acute Q fever is defined as primary infection with *C*. *burnetii*, and <60% of infected patients may be asymptomatic [9]. However, acute Q fever can manifest as a flu-like and self-limited illness, and major clinical presentations of these patients are fever, headache, coughing, atypical pneumonia, hepatitis, myalgia, arthralgia, cardiac involvement, skin rash and neurologic signs, and 2% of patients with acute disease are hospitalized. The case fatality rate of acute Q fever is reported up to 1–2% [4,8,10]. Approximately 5% of acute Q fever cases go on to develop chronic Q fever. People may become chronically infected without having being previously diagnosed with acute disease, and chronic Q fever may manifest months or years after an acute infection [11]. Chronic Q fever is accompanied with endocarditis, vascular infection, prosthetic joint arthritis, osteoarticular infection and lymphadenitis [4,12,13]. Endocarditis and vascular infection caused by Q fever are fatal if untreated[9].

Human Q fever has been described in countries around the world, New Zealand being the only exception. As it is not a notifiable disease in many countries, the geographical distribution of the organism is extrapolated from serological surveys and investigated outbreaks[3]. In Iran, the first clinical cases of acute Q fever are reported in 1952. From 1970 to 1976, 133 patients with acute Q fever were reported from different parts of Iran [14]. After 1976, Q fever was neglected in Iran, and no human case was reported. At the same time with large outbreak of Q fever in the Netherlands (2007–2010)[15],*C*. *burnetii* antibodies were reported in febrile patients in the Kerman province (southeastern Iran), [16]and investigation for Q fever was resumed. After that, various seroepidemiological studies were conducted on animal and human population. The first case of chronic Q fever (endocarditis) was reported in 2013 [17].

We do not have an overall estimation of Q fever infection in Iran. Current studies have reported Q fever seroprevalence in human and domestic animals. The overall estimation of Q fever seroprevalence in the human and animal population will help health policymakers create or modify control and prevention programs for Q fever in Iran. In the present systematic review, we reviewed the local Iranian publications on Q fever and also international publications relating to the disease in Iran. In this report we provide a summary of the more recent data collected on Q fever in Iran.

## Methods

### Information sources and search

From January 2005 to June 2016, we searched the literature for articles that assessed the prevalence of Q fever infection in human and animals in Iran.

We searched multiple English and Persian electronic data sources including Iranmedex, Scientific Information Database (SID), Magiran, Iranian Research Institute for Information Science and Technology (IRANDOC), Google Scholar, Medline, PubMed, Science Direct, Scopus and Web of science. In addition, the citations of the included articles were reviewed to find other relevant studies. We also looked at the electronic abstract list of congress conducted in Iran and also at the electronic database of students’ thesis. Keywords that we used for our search were “Q fever, *Coxiella burnetii* and Iran".

### Eligibility criteria and study selection

Articles with cross sectional design which were sampling from Iran, published in Persian or English and measured seroposivity by serological assays (just IgG) were eligible to enter meta-analysis.

Exclusion criteria for studies from systematic review were: 1- Lack of access to full article or insufficient data in abstract; 2- Unclear testing methods used to detect studied infection or non-serology test 3- IgM detection4- other study design except cross sectional.

We contacted the corresponding author when we have questions about the eligibility of the article.

### Data collection and data items

Data was extracted by two reviewers and checked twice based on the following items: type of study, sample size, location and time of the study, species and prevalence of Q fever. We grouped the studies with species in herd and individual level as sheep, goat, cattle and camel and also human participants as phase I and phase II IgG.

### Analytic approach

We conducted meta-analyses in STATA version 12. We did meta-analysis for Q fever prevalence in any species in herd and individual level and in phase I and II for human. The outcome was measured and reported as prevalence, with point and 95% confidence intervals. A Q-test was used to assess heterogeneity. When the heterogeneity test had a p-value less than 0.1, a random-effects model was used; otherwise the fixed-effects model was used to calculate the pooled prevalence. Also by calculating pooled Q fever seroprevalence in each province we mapped prevalence of Q fever using ArcGIS ver. 10.2.

## Results

### Description of included studies

**Fig 1:**
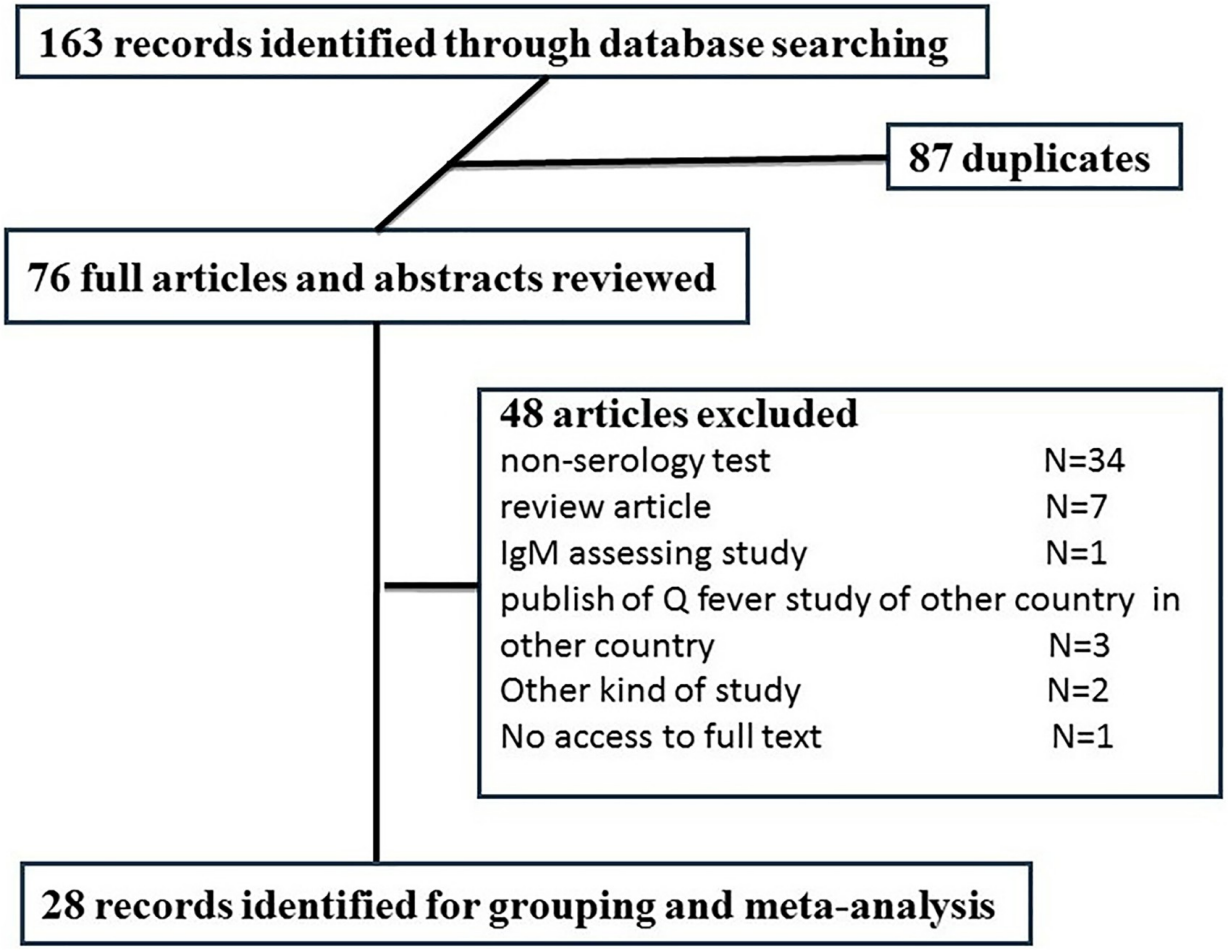
Flow diagram of included and excluded records

As presented in Fig 1, we found 163 abstracts in our literature review. After removing duplications (n = 87) based on title and abstract, 76 remained for full text review. Of those, 48 articles were excluded for various reasons including non-serology test (n = 34), review article (n = 7), IgM assessing study (n = 1), publish of Q fever study of other country in Iranian journals (n = 3), other kind of study (n = 2) and no access to full text (n = 1) (Fig 1). Characteristics of the final included studies (n = 28) in the systematic review showed in Table 1.

**Table 1:** Characteristics of the included studies in the systematic review, 2005–2016.

	Group	Level	Sample Size	Time Study	Province	Reference
1	Human	Phase I	187	2011	Sistan and Baluchistan	[44]
		Phase II				
2	Human	Phase I	75	2009	Kerman	[16]
		Phase II				
3	Human	Phase I	250	2011–2012	Kurdistan	[45]
		Phase II				
4	Human	Phase II	200	2014	Ardabil	[46]
					Khuzestan	
5	Human	Phase II	105	2011	Sistan v Baluchistan	[47]
6	Human	Phase II	45	2013	Kerman	[48]
7	Human	Phase II	75	2010–2011	Kerman	[49]
8	Human	Phase II	121	2014	Kerman	[50]
9	Human	Phase II	53	2015–2016	Mazandaran	[51]
10	Human	Phase II	92	2014	South Khorasan	[52]
11	Sheep	Herd	10	2009	Kerman	[53]
		Individual	85			
12	Sheep	Herd	29	2012	Khorasan Razavi	[54]
		Individual	255			
13	Sheep	Herd	10	2014	Hamadan	[41]
		Individual	200			
14	Sheep	Individual	256	2011–2012	Ardabil	[55]
15	Sheep	Individual	235	2011–2012	Fars	[34]
			336		Isfahan	
			297		Khorasan Razavi	
			232		Markazi	
16	Sheep	Individual	12	2011	Hormozgan	[35]
			37	2011	Kerman	
			78	2011	Sistan v Baluchistan	
17	Sheep	Individual	220	2010–2011	Khuzestan	[56]
18	Sheep	Individual	253	2011–2012	Mazandaran	[57]
19	Goat	Herd	9	2008	Kerman	[58]
		Individual	79			
20	Goat	Herd	28	2012	Khorasan Razavi	[54]
		Individual	205			
21	Goat	Herd	10	2014	Hamadan	[41]
		Individual	50			
22	Goat	Individual	76	2011–2012	Fars	[34]
			76		Isfahan	
			13		Khorasan Razavi	
			15		Markazi	
23	Goat	Individual	58	2011	Kerman	[59]
24	Goat	Individual	39	2011	Hormozgan	[35]
			136		Kerman	
			66		Sistan v Baluchistan	
25	Cattle	Herd	10	2014	Hamedan	[41]
		Individual	120			
26	Cattle	Herd	12	2008	Kerman	[58]
		Individual	93			
27	Cattle	Herd	44	2010	Kerman	[60]
28	Cattle	Herd	19	2011	Kerman	[40]
		Individual	161			
29	Cattle	Herd	19	2010	Khorasan Razavi	[61]
		Individual	246			
30	Cattle	Herd	34	2014	Kurdistan	[62]
31	Cattle	Herd	37	2014	West Azerbaijan	[63]
32	Cattle	Individual	86	2011	Khuzestan	[64]
33	Camel	Individual	42	2012–2013	Khorasan Razavi	[65]
			59		North Khorasan	
			66		South Khorasan	
34	Dog	Individual	182	2013–2014	Khuzestan	[66]

### Prevalence of seropositivity

#### Q fever seroprevalence in human

**Fig 2:**
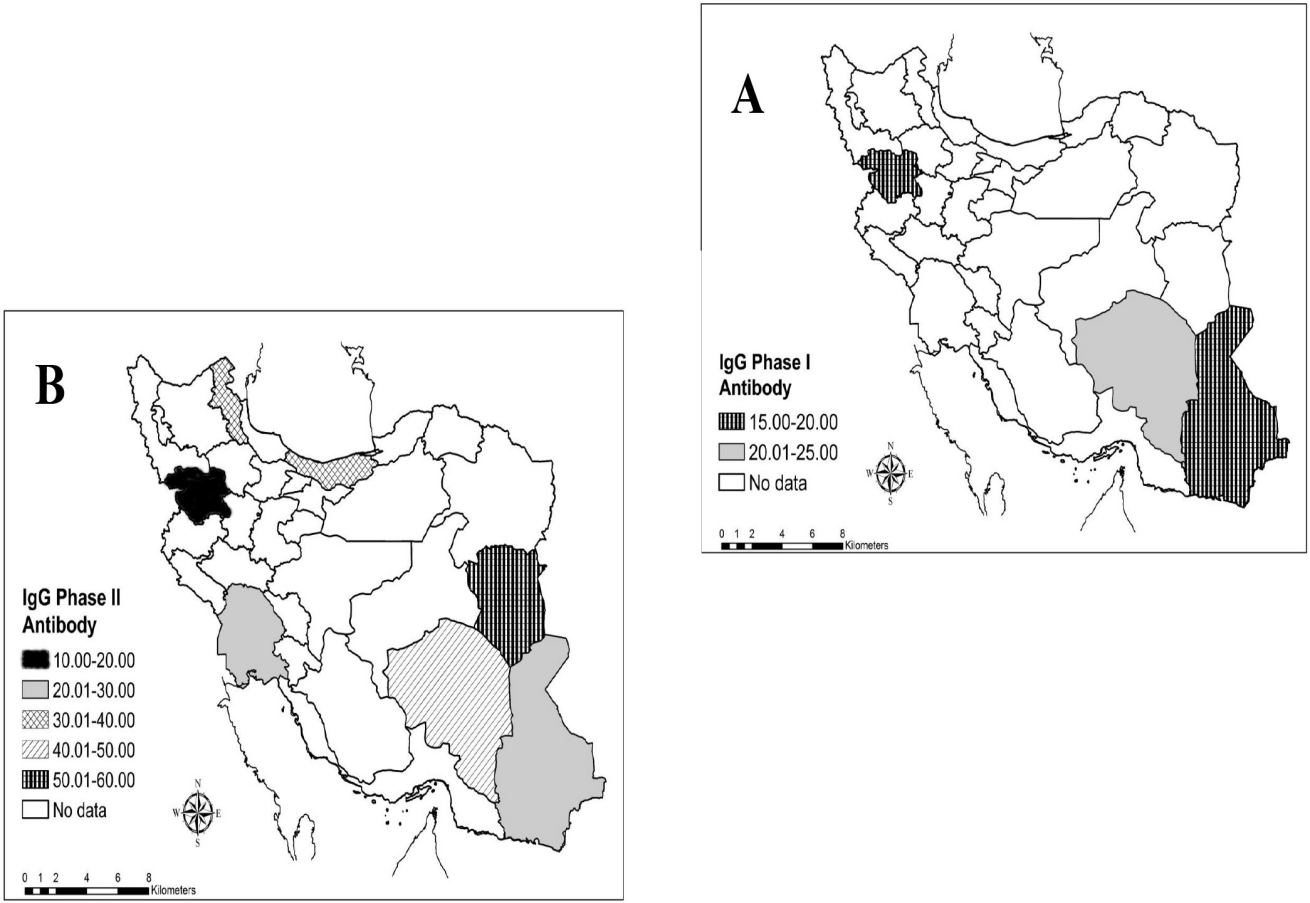
Geographical distribution of anti- *C*. *burnetii* IgG Phase I (A) and IgG Phase II (B) among Iranian people.

In final, 10 studies were found about seroprevalence of Q fever in different parts of Iran which three studies were about IgG phase I antibody and eighth studies were IgG phase II antibody. The overall seroprevalence of IgG phase I and II antibodies of Q fever in human was 19.80% (95% CI: 16.35–23.25%) and 32.86% (95% CI: 23.80–41.92%), respectively (Table 2). Geographical distribution of Q fever seropositivity was shown in Fig 2. *C*. *burnetii* antibodies have been detected in human from 9 provinces. The most prevalence of IgG phases I and II antibodies was seen in Kerman (24%) and South Khorasan (54%) provinces, respectively (Fig 2).

**Table 2:** Prevalence Q fever antibody among human and domestic animals, 2005–2016

	Level	Sample size	Number of studies	Pooled estimate (%)
**Human**	Phase I	512	3	19.80 (16.35–23.25)
	Phase II	1203	10	32.86 (23.80–41.92)
**Goat**	Herd	47	3	93.42(80.23–100.00)
	Individual	813	6	31.97 (20.96–42.98)
**Sheep**	Herd	49	3	96.07 (89.11–100.00)
	Individual	2496	9	24.66 (19.81–29.51)
**Cattle**	Herd	175	7	41.37 (17.88–64.86)
	Individual	706	5	13.30 (2.98–23.62)
**Camel**	Individual	167	1	28.26 (21.47–35.05)
**Dog**	Individual	182	1	0.55 (0.03–2.68)

#### Q fever seroprevalence in goat

**Fig 3:**
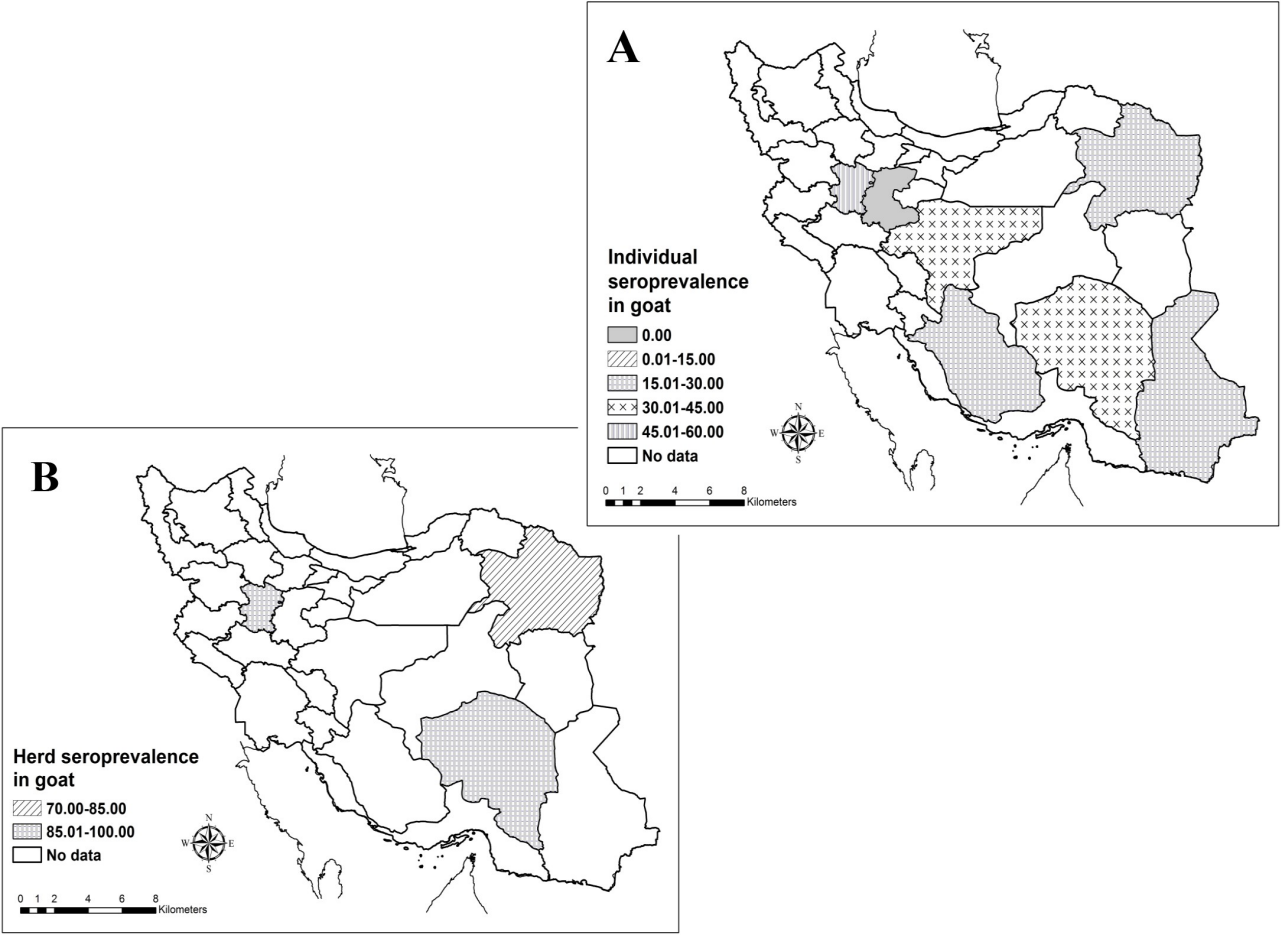
Geographical distribution of Q fever seropositivity among goats in individual (A) and herd (B) levels in the Iran.

Six studies were conducted about seroprevalence of Q fever in goats which three studies were in herd level and six studies were in individual’s level. The herd and individual prevalence of Q fever antibody in goat were 93.42% (95% CI: 80.23–100.00) and 31.97% (95% CI: 20.96–42.98%), respectively. The higher and lower seroprevalence was seen in Kerman (63.3%) and Markazi (0%) provinces, respectively. Also seroprevalence of Q fever among goats in Iran showed Fig 3.

#### Q fever seroprevalence in sheep

**Fig 4:**
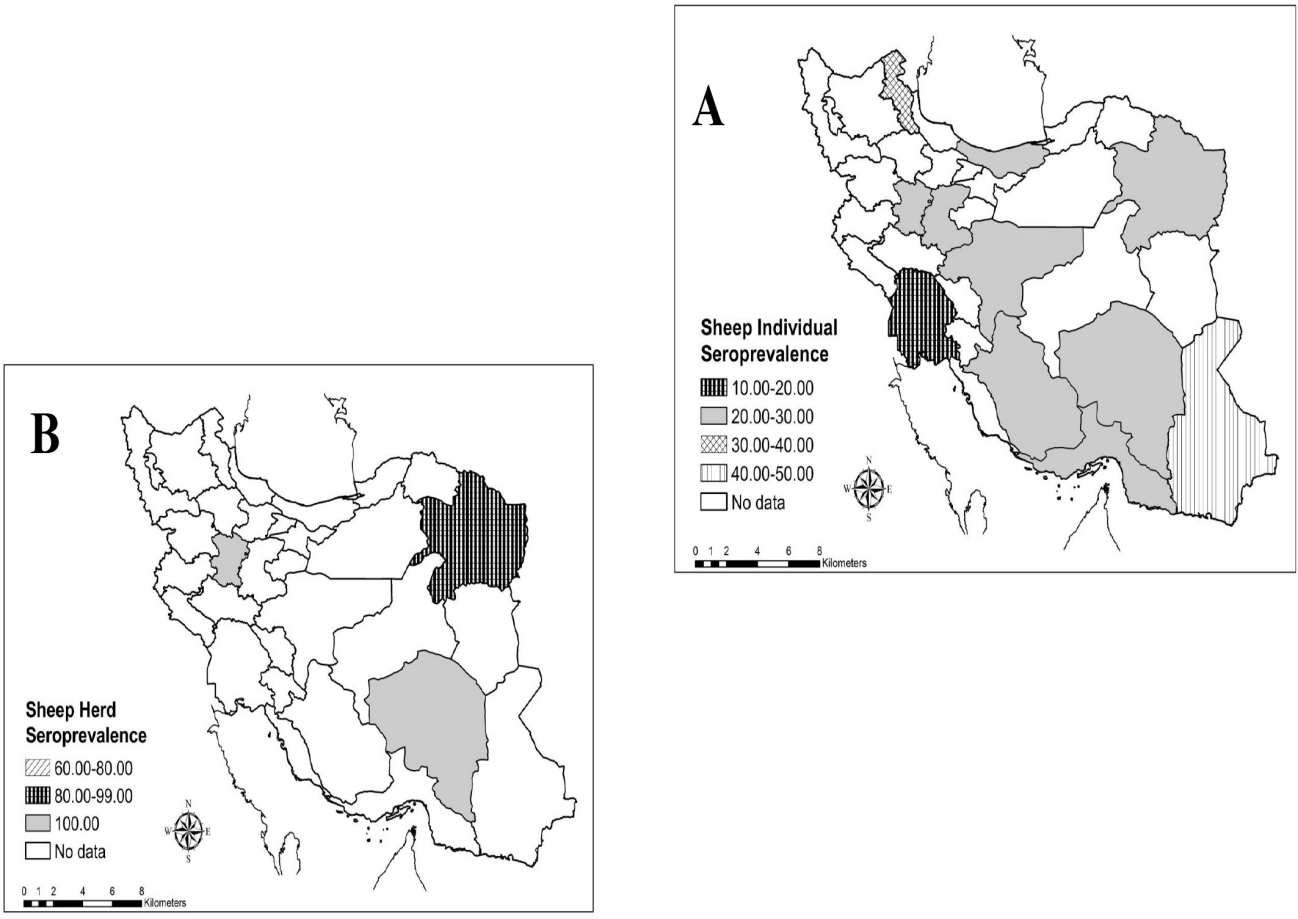
Geographical distribution of *C*. *burnetii* seroprevalence among sheep’s in individual (A) and herd (B) levels in Iran.

In final, 8 studies were found about seroprevalence of Q fever in sheep’s, which 3 studies were in herd level and 9 studies were in individual’s level. The herd and individual prevalence of Q fever antibody in sheep’s were 96.07% (95% CI: 89.11–100.00%) and 24.66% (95% CI: 19.81–29.51%), respectively. The higher and lower seropositivity of *C*. *burnetii* among sheep’s showed in Sistan va Baluchestan (43.6%) and Khorasan Razavi (12.8%) provinces, respectively. Also geographical distribution of Q fever seropositivity in sheep’s was shown in Fig 4.

#### Q fever seroprevalence in cattle

**Fig 5:**
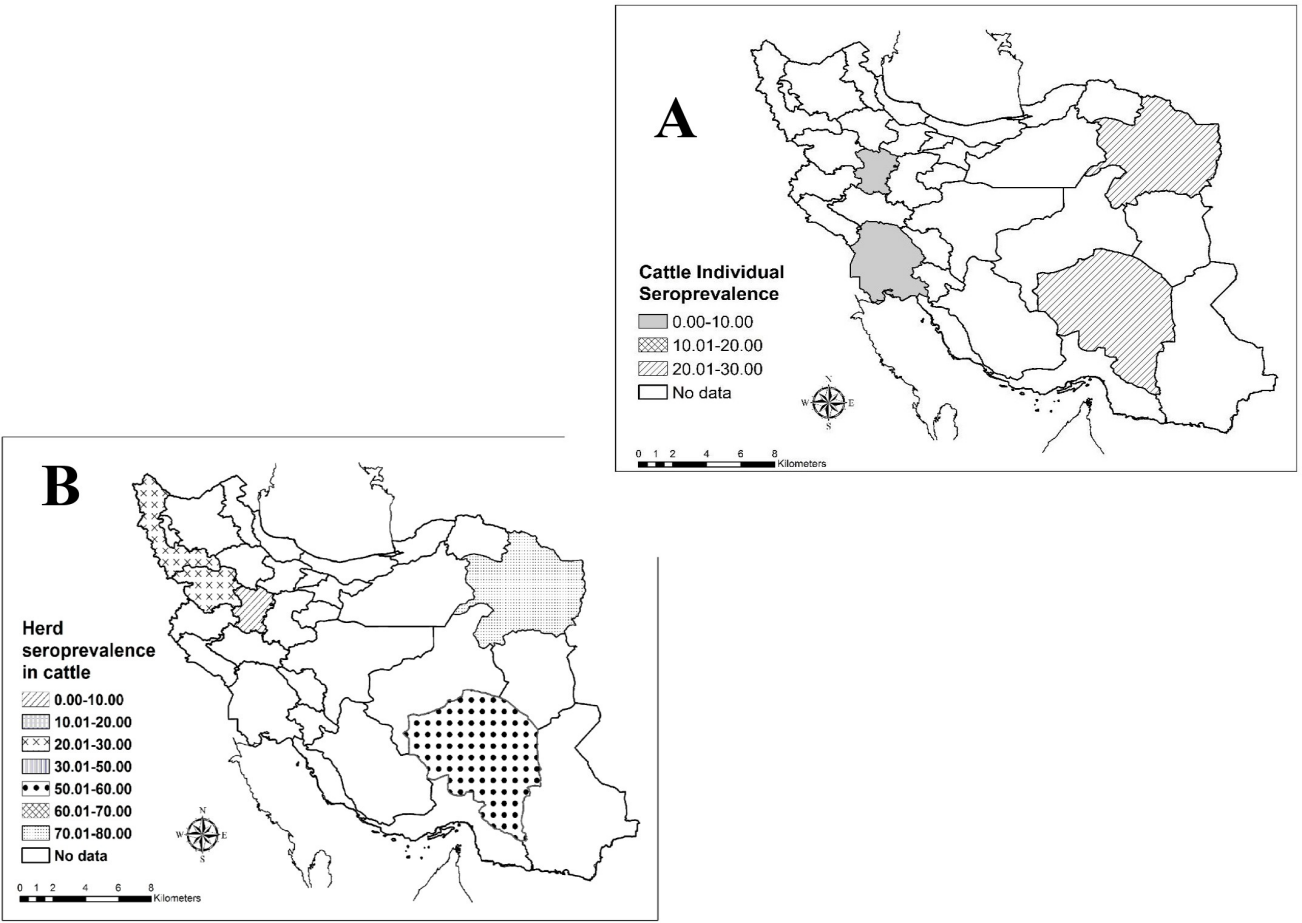
Geographical distribution of Q fever seropositivity among cattle’s in individual (A) and herd (B) levels in Iran.

In final, 8 studies had been done about seroprevalence of Q fever in sheep’s, which 7 studies were in herd level and 5 studies were in individual’s level. The herd and individual prevalence of Q fever antibody in cattle were 41.37% (95% CI: 17.88–64.86%) and 13.30% (95% CI: 2.98–23.62%), respectively. The seroprevalence of Q fever among cattle’s in the different parts of Iran showed in Fig 5.

#### Other animals

Only one study found the seroprevalence of dogs and camels in Iran. Individual seropositivity of Q fever in camel and dog were 28.26% (95% CI: 21.47–35.05) and 0.55% (0.03–2.68), respectively.

## Discussion

The current systematic review reports the seroprevalence of Q fever among human and domestic animals in Iran. The results of this meta-analysis showed the prevalence of IgG phase I and II antibodies of *C*. *burnetii* among human in Iran were 19.80% and 32.86%, respectively. These rates were very high compared with other similar study. As example seropositivity of Q fever in China was reported 10% [18]. In a recent systematic review study, human seroprevalence was reported from 1–32% in Africa [19]. Human seroprevalence of Q fever were reported 3 to 35.8% in Kenya [20], 12.3–32%in Turkey[21,22], 15.3% in Spain [23],5.2%in Australia[24]and 11% in Denmark[25]. Overall seroprevalence for *C*. *burnetii* was reported 3.1% among general population in the USA [26]. The differences between countries could be due to varieties in ecologic, social, cultural, behavioral and economical conditions and also levels of animals infections, which affect the exposures of people in each of the regions of the world. In all conducted studies in Iran, *C*. *burnetii* antibodies have been detected in human from 9 provinces. Seroprevalence varied in different areas of Iran. The seroprevalence of IgG phase I of Q fever ranged from 18.2% to 24%, which Sistan and Baluchestan and Kerman provinces had lowest and highest seropositivity, respectively. Also the prevalence of IgG phase II antibodies ranged from 14.5% to 68%, which higher and lower seroprevalence showed in Kerman and Sistan and Baluchestan provinces, respectively [17,18]. According to the findings of this study, it is highly recommended that physicians and health care workers are informed about bacteria circulating in Iran.

Goats are important sources of *C*. *burnetii* infection in people and seven serosurveys were conducted on goats in Iran between 2005 and 2016. According to the results of this meta-analysis, the seroprevalence of Q fever in goats was 31.97%. Also, 93.42% of the goat’s herds were seropositive in Iran. In similar studies, goats seropositivity were 13% to 23% in Africa [19],20% to 46% in Kenya [20] and 0.8% to 60.6% in China [18]. Antibodies against *C*. *burnetii* in Netherland, Bulgaria and Bangladesh were 7.8%, 13.7% and 9.52%, respectively [27–29]. Our study showed *C*. *burnetii* antibodies have been detected in goats from 7 provinces. The higher and lower seroprevalence was seen in Kerman (65.8%) and Markazi (0%) provinces, respectively. From 2007 to 2010, more than 4,000 human cases were diagnosed in Netherlands. The outbreaks in humans were mainly related area with intensive dairy goat farming [15]. A recent (2012–2014) human outbreak of Q fever in the Australia was linked to an intensive goat and sheep dairy farm and also seroprevalence in goats was 15% [30]. Due to the recent outbreaks of Q fever, it seems that the goat is very strong role in human infections. In Iran, due to the high seroprevalence antibodies to *C*. *burnetii* in goats, this case can be possible, but more studies are needed to prove this point in the future.

Large human Q fever outbreaks related to sheep in published studies around the world included Bulgaria (2009), Croatia (2008),France (2007), Germany (2006, 2008), Italy (2004) and Switzerland (1987) [31]. Therefore, sheep is considered as an important factor for human Q fever infection. The results of this meta-analysis demonstrated that were 24.66% of sheep’s had antibody of *C*. *burnetii* in Iran. In total, seroprevalence of Q fever was 96.07% among sheep’s herds. Different rates of sheep seroprevalence were reported in other countries, so that the seropositivity was 2.4% in Netherlands [32], 5% in China [18], 11.6% in Bulgaria [28], 20% in Turkey [33] and 11% to 33% in Africa [19]. The studies of Q fever seroprevalence found in 12 provinces in Iran. The higher and lower seropositivity of *C*. *burnetii* among sheep’s showed in Sistan and Baluchestan (43.6%) and Khorasan Razavi (12.8%) provinces, respectively [34,35].

In cattle's like other main reservoirs (sheep and goat) of Q fever, *C*. *burnetii* is shed by birth products (placenta, birth fluids), but may also be shed by vaginal mucus, milk, and faeces, urine and semen[36].Contact with these contaminated materials can lead to human infection. According to the results of this meta-analysis, the seroprevalence of Q fever in cattle was 13.30%. Also, 41.37% of the cattle’s herds were seropositive in Iran. In other countries, seroprevalence of Q fever was different rates among cattle, for example: 6.2% in Northern Ireland[37], 8.5% in in Bulgaria [28], 15% in China [18], 16.0% in Netherlands [38] and 30.4% in Cameroon [39]. In all conducted studies in Iran, *C*. *burnetii* antibodies have been detected in human from 7 provinces. The higher and lower seroprevalence of Q fever was seen in Kerman (29.2%) and Hamadan (0.8%) provinces, respectively[40,41]. The lower seroprevalence in cattle’s compared to the two other main reservoirs (sheep and goat) in Iran, this can be caused by difference in strains circulating in Iran and other areas and genotyping studies can be helpful in support of this subject. Therefore, it seems that there are differences in geographic prevalence of the disease among cattle in Iran and it is recommended to be done in the future a comprehensive study to determine the case in all provinces of Iran.

The seropositivity of Q fever among camel was 28.26% in Iran. Antibodies to *C*. *burnetii* were reported in 51.6% and 80% of camels in Saudi Arabia and Chad, respectively [42,43]. Also, the seroprevalence of Q fever was 0.55% among dogs in Iran, but only one study conducted in Iran. For better judgment on this issue needs more studies in the future.

Although human and animal infections of Q fever are known to occur and endemic in Iran, but the Q fever is not a reportable disease in the country and clinical cases are probably largely unrecognizable by health system. There is a need for information on the epidemiology of *C*. *burnetii* in Iran as well as many other issues such as distribution, pathogenesis and molecular typing. The data from the studies to date in Iran provide only a basic picture of Q fever in the country. Active case finding and further research studies are recommended, to more clearly define the epidemiology and importance of *C*. *burnetii* infections in animals and people in Iran. This will enable the formulation and implementation of locally applicable control methods for Q fever which can be implemented by animal and human healthcare workers.

### Ethical approval

Not applicable.

### Informed consent

Not applicable.

## Supporting information

S1 Fig PRISMA 2009 Flow Diagram. (PDF)

S1 Table PRISMA 2009 Checklist. (DOC)

## References

[1] van Schaik EJ, Chen C, Mertens K, Weber MM, Samuel JE (2013) Molecular pathogenesis of the obligate intracellular bacterium Coxiella burnetii. Nature Reviews Microbiology 11: 561–573. doi: 10.1038/nrmicro3049 23797173 10.1038/nrmicro3049 PMC4134018

[2] Madariaga MG, Rezai K, Trenholme GM, Weinstein RA (2003) Q fever: a biological weapon in your backyard. The Lancet infectious diseases 3: 709–721. 14592601 10.1016/s1473-3099(03)00804-1

[3] Oyston P, Davies C (2011) Q fever: the neglected biothreat agent. Journal of medical microbiology 60: 9–21. doi: 10.1099/jmm.0.024778-0 21030501 10.1099/jmm.0.024778-0

[4] Angelakis E, Raoult D (2010) Q fever. Veterinary microbiology 140: 297–309. doi: 10.1016/j.vetmic.2009.07.016 19875249 10.1016/j.vetmic.2009.07.016

[5] Raoult D, Marrie T, Mege J (2005) Natural history and pathophysiology of Q fever. The Lancet infectious diseases 5: 219–226. doi: 10.1016/S1473-3099(05)70052-9 15792739 10.1016/S1473-3099(05)70052-9

[6] Bouvery NA, Souriau A, Lechopier P, Rodolakis A (2003) Experimental Coxiella burnetii infection in pregnant goats: excretion routes. Veterinary research 34: 423–433. doi: 10.1051/vetres:2003017 12911859 10.1051/vetres:2003017

[7] Tissot-Dupont H (2004) Wind in November, Q fever in December. Emerging Infectious Disease 10: 1264–1269. 10.3201/eid1007.030724 PMC3323349 15324547

[8] Parker NR, Barralet JH, Bell AM (2006) Q fever. The Lancet 367: 679–688. 10.1016/S0140-6736(06)68266-4 16503466

[9] Anderson A, Bijlmer H, Fournier P-E, Graves S, Hartzell J, et al (2013) Diagnosis and management of Q fever—United States, 2013: recommendations from CDC and the Q Fever Working Group. MMWR Recomm Rep 62: 1–30. 23535757

[10] Frankel D, Richet H, Renvoisé A, Raoult D (2011) Q fever in France, 1985–2009. Emerg Infect Dis 17: 350–356. doi: 10.3201/eid1703.100882 21392423 10.3201/eid1703.100882 PMC3166002

[11] Fenollar F, Fournier P-E, Carrieri MP, Habib G, Messana T, et al (2001) Risks factors and prevention of Q fever endocarditis. Clinical Infectious Diseases 33: 312–316. doi: 10.1086/321889 11438895 10.1086/321889

[12] Raoult D (2012) Chronic Q fever: expert opinion versus literature analysis and consensus. Journal of Infection 65: 102–108. doi: 10.1016/j.jinf.2012.04.006 22537659 10.1016/j.jinf.2012.04.006

[13] Eldin C, Mélenotte C, Mediannikov O, Ghigo E, Million M, et al (2017) From Q fever to Coxiella burnetii infection: a paradigm change. Clinical Microbiology Reviews 30: 115–190. doi: 10.1128/CMR.00045-16 27856520 10.1128/CMR.00045-16 PMC5217791

[14] Mostafavi E, Rastad H, Khalili M (2012) Q fever: an emerging public health concern in Iran. Asian Journal of Epidemiology 5: 66–74.

[15] Dijkstra F, van der Hoek W, Wijers N, Schimmer B, Rietveld A, et al (2012) The 2007–2010 Q fever epidemic in The Netherlands: characteristics of notified acute Q fever patients and the association with dairy goat farming. FEMS Immunology & Medical Microbiology 64: 3–12. 22066649 10.1111/j.1574-695X.2011.00876.x

[16] Khalili M, Shahabi-Nejad N, Golchin M (2010) Q fever serology in febrile patients in southeast Iran. Transactions of the Royal Society of Tropical Medicine and Hygiene 104: 623–624. doi: 10.1016/j.trstmh.2010.04.002 20627331 10.1016/j.trstmh.2010.04.002

[17] Yaghmaie F, Esmaeili S, Francis SA, Mostafavi E (2015) Q fever endocarditis in Iran: A case report. Journal of infection and public health 8: 498–501. doi: 10.1016/j.jiph.2014.12.004 25747823 10.1016/j.jiph.2014.12.004

[18] El-Mahallawy H, Lu G, Kelly P, Xu D, Li Y, et al (2015) Q fever in China: a systematic review, 1989–2013. Epidemiology and infection 143: 673–681. doi: 10.1017/S0950268814002593 25274488 10.1017/S0950268814002593 PMC9507106

[19] Vanderburg S, Rubach MP, Halliday JE, Cleaveland S, Reddy EA, et al (2014) Epidemiology of Coxiella burnetii infection in Africa: a OneHealth systematic review. PLoS Negl Trop Dis 8: e2787 doi: 10.1371/journal.pntd.0002787 24722554 10.1371/journal.pntd.0002787 PMC3983093

[20] Njeru J, Henning K, Pletz M, Heller R, Neubauer H (2016) Q fever is an old and neglected zoonotic disease in Kenya: a systematic review. BMC public health 16: 1. 27048480 10.1186/s12889-016-2929-9 PMC4822290

[21] Gozalan A, Rolain J, Ertek M, Angelakis E, Coplu N, et al (2010) Seroprevalence of Q fever in a district located in the west Black Sea region of Turkey. European journal of clinical microbiology & infectious diseases 29: 465–469. 20195671 10.1007/s10096-010-0885-3

[22] Kilic S, Yilmaz GR, Komiya T, Kurtoglu Y, Karakoc EA (2008) Prevalence of Coxiella burnetii antibodies in blood donors in Ankara, Central Anatolia, Turkey. New Microbiol 31: 527–534. 19123309

[23] Cardenosa N, Sanfeliu I, Font B, Munoz T, Nogueras MM, et al (2006) Seroprevalence of human infection by Coxiella burnetii in Barcelona (northeast of Spain). The American journal of tropical medicine and hygiene 75: 33–35. 16837705

[24] Tozer S, Lambert S, Sloots T, Nissen M (2011) Q fever seroprevalence in metropolitan samples is similar to rural/remote samples in Queensland, Australia. European journal of clinical microbiology & infectious diseases 30: 1287–1293. 21499708 10.1007/s10096-011-1225-y

[25] Bosnjak E, Hvass A, Villumsen S, Nielsen H (2010) Emerging evidence for Q fever in humans in Denmark: role of contact with dairy cattle. Clinical microbiology and infection 16: 1285–1288. doi: 10.1111/j.1469-0691.2009.03062.x 19832723 10.1111/j.1469-0691.2009.03062.x

[26] Anderson AD, Kruszon-Moran D, Loftis AD, McQuillan G, Nicholson WL, et al (2009) Seroprevalence of Q fever in the United States, 2003–2004. The American journal of tropical medicine and hygiene 81: 691–694. doi: 10.4269/ajtmh.2009.09-0168 19815888 10.4269/ajtmh.2009.09-0168

[27] Van den Brom R, Moll L, Van Schaik G, Vellema P (2013) Demography of Q fever seroprevalence in sheep and goats in The Netherlands in 2008. Preventive veterinary medicine 109: 76–82. doi: 10.1016/j.prevetmed.2012.09.002 23031327 10.1016/j.prevetmed.2012.09.002

[28] Martinov S (2007) Contemporary state of the problem Q fever in Bulgaria. Biotechnology & Biotechnological Equipment 21: 353–361.

[29] Rahman MA, Alam MM, Islam MA, Bhuiyan A, Rahman A (2016) Serological and Molecular Evidence of Q Fever in Domestic Ruminants in Bangladesh. Veterinary Medicine International 2016. 10.1155/2016/9098416 PMC4867074 27239369

[30] Bond K, Vincent G, Wilks C, Franklin L, Sutton B, et al (2016) One Health approach to controlling a Q fever outbreak on an Australian goat farm. Epidemiology and infection 144: 1129–1141. doi: 10.1017/S0950268815002368 26493615 10.1017/S0950268815002368 PMC4825098

[31] Van den Brom R, van Engelen E, Roest H, van der Hoek W, Vellema P (2015) Coxiella burnetii infections in sheep or goats: an opinionated review. Veterinary microbiology 181: 119–129. doi: 10.1016/j.vetmic.2015.07.011 26315774 10.1016/j.vetmic.2015.07.011

[32] Van den Brom R, Vellema P (2009) Q fever outbreaks in small ruminants and people in the Netherlands. Small Ruminant Research 86: 74–79.

[33] Kennerman E, Rousset E, Gölcü E, Dufour P (2010) Seroprevalence of Q fever (coxiellosis) in sheep from the Southern Marmara Region, Turkey. Comparative immunology, microbiology and infectious diseases 33: 37–45. doi: 10.1016/j.cimid.2008.07.007 18848356 10.1016/j.cimid.2008.07.007

[34] Asadi J, Kafi M, Khalili M (2013) Seroprevalence of Q fever in sheep and goat flocks with a history of abortion in Iran between 2011 and 2012. Vet Ital 49: 163–168. 23888416

[35] Ezatkhah M, Alimolaei M, Khalili M, Sharifi H (2015) Seroepidemiological study of Q fever in small ruminants from Southeast Iran. Journal of infection and public health 8: 170–176. doi: 10.1016/j.jiph.2014.08.009 25270385 10.1016/j.jiph.2014.08.009

[36] Guatteo R, Beaudeau F, Berri M, Rodolakis A, Joly A, et al (2006) Shedding routes of Coxiella burnetii in dairy cows: implications for detection and control. Veterinary Research 37: 827–833. doi: 10.1051/vetres:2006038 16973121 10.1051/vetres:2006038

[37] McCaughey C, Murray L, McKenna J, Menzies F, McCullough S, et al (2010) Coxiella burnetii (Q fever) seroprevalence in cattle. Epidemiology and infection 138: 21–27. doi: 10.1017/S0950268809002854 19480726 10.1017/S0950268809002854

[38] Muskens J, Van Engelen E, Van Maanen C, Bartels C, Lam T (2011) Prevalence of Coxiella burnetii infection in Dutch dairy herds based on testing bulk tank milk and individual samples by PCR and ELISA. The veterinary record 168: 79 doi: 10.1136/vr.c6106 21257587 10.1136/vr.c6106

[39] Scolamacchia F, Handel IG, Fèvre EM, Morgan KL, Tanya VN, et al (2010) Serological patterns of brucellosis, leptospirosis and Q fever in Bos indicus cattle in Cameroon. PLoS One 5: e8623 doi: 10.1371/journal.pone.0008623 20098670 10.1371/journal.pone.0008623 PMC2809085

[40] Khalili M, Sakhaee E, Babaei H (2012) Frequency of anti-Coxiella burnetii antibodies in cattle with reproductive disorders. Comparative clinical pathology 21: 917–919.

[41] Edalati-Shokat H, Abbasi-Doulatshahi E, Hajian-Bidar H, Gharekhani J, Rezaei A-A (2015) Q fever in domestic ruminants: A Seroepidemiological survey in Hamedan, Iran. Int J Curr Microbiol App Sci 4: 589–596.

[42] Hussein MF, Alshaikh MA, Al-Jumaah RS, GarelNabi A, Al-Khalifa I, et al (2015) The Arabian camel (Camelus dromedarius) as a major reservoir of Q fever in Saudi Arabia. Comparative Clinical Pathology 24: 887–892.

[43] Schelling E, Diguimbaye C, Daoud S, Nicolet J, Boerlin P, et al (2003) Brucellosis and Q-fever seroprevalences of nomadic pastoralists and their livestock in Chad. Preventive veterinary medicine 61: 279–293. 14623412 10.1016/j.prevetmed.2003.08.004

[44] Esmaeili S, Naddaf SR, Pourhossein B, Shahraki AH, Amiri FB, et al (2016) Seroprevalence of Brucellosis, Leptospirosis, and Q Fever among Butchers and Slaughterhouse Workers in South-Eastern Iran. PloS one 11: e0144953 doi: 10.1371/journal.pone.0144953 26731333 10.1371/journal.pone.0144953 PMC4701462

[45] Esmaeili S, Pourhossein B, Gouya MM, Amiri FB, Mostafavi E (2014) Seroepidemiological survey of Q fever and brucellosis in Kurdistan Province, western Iran. Vector-Borne and Zoonotic Diseases 14: 41–45. doi: 10.1089/vbz.2013.1379 24359427 10.1089/vbz.2013.1379 PMC3880925

[46] Khayyat KM, Asadi J, Khalili M, Abiri Z (2016) The First Serological Study of Coxiella burnetii among Pregnant Women in Iran. Iranian journal of public health 45: 523 27252922 PMC4888180

[47] Metanat M, RAD NS, Alavi-Naini R, Shahreki S, Sharifi-Mood B, et al (2014) Acute Q fever among febrile patients in Zahedan, southeastern Iran. Turkish journal of medical sciences 44: 99–103. 25558567 10.3906/sag-1209-102

[48] Naderipour Z, Golchin M, Khalili M (2014) Design of an ELISA kit for detection human acute Q fever. Iranian Journal of Medical Microbiology 8: 28–34.

[49] Khalili M, Mosavi M, Diali HG, Mirza HN (2014) Serologic survey for Coxiella burnetii phase II antibodies among slaughterhouse workers in Kerman, southeast of Iran. Asian Pacific journal of tropical biomedicine 4: S209–S212. doi: 10.12980/APJTB.4.2014C1268 25183082 10.12980/APJTB.4.2014C1268 PMC4025290

[50] Khalili M, Qorbani A, Sharifi H, Golchin M (2015) Prevalence and risk factor of Q fever among veterinary students in Iran. Tropical Biomedicine 32: 704–709. 33557462

[51] Ghasemian R, Mostafavi E, Esmaeili S, Najafi N, Arabsheybani S (2016) A Survey of Acute Q Fever among Patients with Brucellosis-Like and Atypical Pneumonia Symptoms Who Are Referred to Qaemshahr Razi Hospital in Northern Iran (2014–2015). Global Journal of Health Science 9: 225–232.

[52] Khalili M, Aflatoonian MR, Rahanjam M, Golchin M, Sharifi H, et al (2016) Frequency of Seropositivity for anti-Coxiella Burnetii (Phase II) among Veterinary Staff in Southern Khorasan, Iran, in 2014. Journal of Kerman University of Medical Sciences 23: 164–173.

[53] Sakhaee E, Khalili M (2010) The first serologic study of Q fever in sheep in Iran. Tropical animal health and production 42: 1561–1564. doi: 10.1007/s11250-010-9606-2 20521106 10.1007/s11250-010-9606-2

[54] Keyvani Rad N, Azizzadeh M, Taghavi Razavizadeh A, Mehrzad J, Rashtibaf M (2014) Seroepidemiology of coxiellosis (Q fever) in sheep and goat populations in the northeast of Iran. Iranian Journal of Veterinary Research 15: 1–6.

[55] Esmaeili S, Bagheri Amiri F, Mostafavi E (2014) Seroprevalence survey of Q fever among sheep in northwestern Iran. Vector-Borne and Zoonotic Diseases 14: 189–192. doi: 10.1089/vbz.2013.1382 24575713 10.1089/vbz.2013.1382

[56] PourMahdi M, Gharibi D, Gorani-Nejad S, Zamiri S (2013) Seroprevalence of coxiellosis in Ahvaz sheep. Iranian Veterinary Journal 9: 11–18.

[57] Esmaeili S, Mostafavi E, Shahdordizadeh M, Mahmoudi H (2013) A seroepidemiological survey of Q fever among sheep in Mazandaran province, northern Iran. Annals of Agricultural and Environmental Medicine 20. 24364439

[58] Khalili M, Sakhaee E (2009) An update on a serologic survey of Q fever in domestic animals in Iran. The American journal of tropical medicine and hygiene 80: 1031–1032. 19478271

[59] Banavand R (2012) Detection of antibodies against to Coxiella burnetii in goat milk samples. Kerman: Shahid Bahoonar.

[60] Khalili M, Sakhaee E, Aflatoonian MR, Shahabi-Nejad N (2011) Herd–prevalence of Coxiella burnetii (Q fever) antibodies in dairy cattle farms based on bulk tank milk analysis. Asian Pacific journal of tropical medicine 4: 58–60. doi: 10.1016/S1995-7645(11)60033-3 21771417 10.1016/S1995-7645(11)60033-3

[61] Azizzadeh M, Mohammadi GR, Haghparast AR, Heidarpour-Bami M (2014) Seroepidemiology of Coxiella Burnetii in commercial dairy herds in northeast of Iran. The Iranian Journal of Veterinary Science and Technology 3: 33–40.

[62] Shajiee A (2014) Prevalence of antibodies against Coxiella burnetii (Q fever) in dairy herds of Sanandaj. Kerman: Shahid Bahoonar

[63] Akbari S (2013) Prevalence of Coxiella burnetii antibodies in Miandoab dairy herds Kerman: Shahid Bahoonar.

[64] Alipour Z (2011) The prevalence of Q fever in dairy cows referred to the veterinary hospital in Ahvaz by ELISA and polymerase chain reaction. Ahvaz: Shahid Chamran.

[65] Janati Pirouz H, Mohammadi G, Mehrzad J, Azizzadeh M, Shirazi MHN (2015) Seroepidemiology of Q fever in one-humped camel population in northeast Iran. Tropical animal health and production 47: 1293–1298. doi: 10.1007/s11250-015-0862-z 26070292 10.1007/s11250-015-0862-z

[66] Rezaei A, Gharibi D, Pourmahdi Borujeni M, Mosallanejad B (2016) Seroprevalence of Lyme disease and Q fever in referred dogs to Veterinary Hospital of Ahvaz. Iranian Veterinary Journal 11: 34–41.

