# Supplementary material for: Seroprevalence of Q fever among human and animal in Iran; A systematic review and meta-analysis
Source: PLoS Negl Trop Dis. 2017 Apr 10;11(4):e0005521. doi: 10.1371/journal.pntd.0005521 (PMC5398711; doi:10.1371/journal.pntd.0005521)
Supplement: S1 Fig — (PDF) [file pntd.0005521.s001.pdf]

PRISMA 2009 Flow Diagram

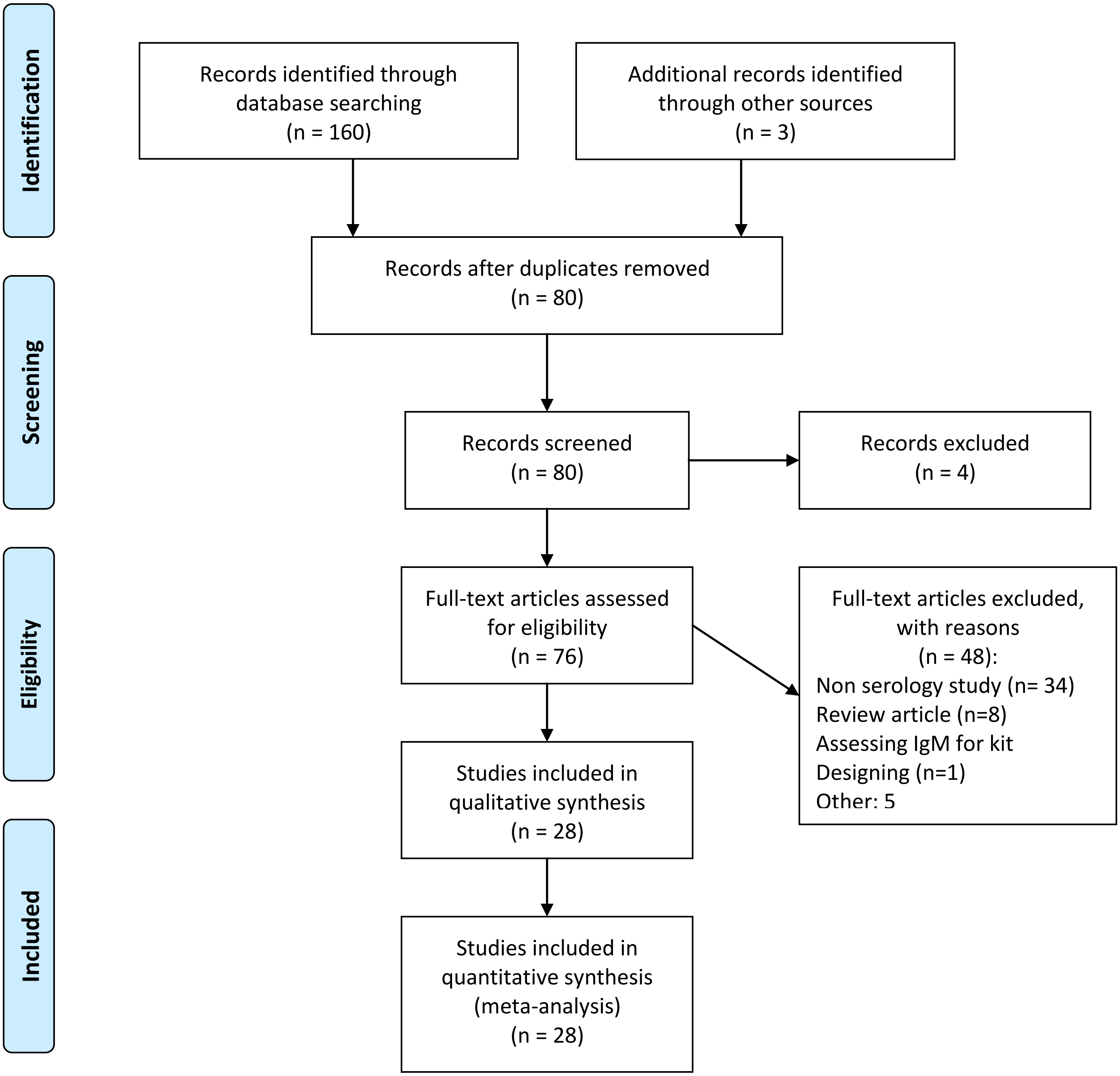

From: Moher D, Liberati A, Tetzlaff J, Altman DG, The PRISMA Group (2009). *P*referred *R*eporting *I*tems for *S*ystematic Reviews and *M*eta-*A*nalyses: The PRISMA Statement. PLoS Med 6(6): e1000097. doi:10.1371/journal.pmed1000097

For more information, visit www.prisma-statement.org.
